# Immune Checkpoint Inhibitor Rechallenge After Prior Immune Toxicity

**DOI:** 10.1007/s11864-022-00995-9

**Published:** 2022-07-25

**Authors:** Sophia Bylsma, Karen Yun, Sandip Patel, Michael J Dennis

**Affiliations:** Department of Medical Oncology, University of California San Diego, 3855 Health Sciences Dr. # 0829, La Jolla, CA 92093-0829 USA

**Keywords:** Immunotherapy, Rechallenge, Immune-related adverse event, PD-1, PD-L1, CTLA-4

## Abstract

Immune checkpoint inhibitors (ICIs) have become an essential part of treatment for many cancer types. These monoclonal antibodies remove a critical negative regulatory signal that allows the immune system to recognize and destroy malignant cells that were previously undetectable. Unfortunately, their use has ushered in a whole new form of drug toxicity whereby the immune system attacks normal tissues in the body, referred to hereafter as immune-related adverse events (irAEs). irAEs are common and can result in treatment discontinuation, hospitalization, and death. When alternative modes of treatment are limited, or considered less efficacious, there may be a desire to resume treatment with ICIs after an irAE. Rechallenge with ICIs carries with it a heightened risk of subsequent toxicity, but with careful consideration and appropriate patient selection, this can be considered a reasonable approach.

## Introduction

The number of patients with cancer who are being treated with immunotherapy is growing rapidly due to the potential benefit of durable responses, even in the setting of metastatic disease [1]. Immune checkpoint inhibitors (ICIs) have emerged as one of the primary treatment modalities for metastatic cancer, as monotherapy or in combination with other systemic agents, such as chemotherapy, monoclonal antibodies, targeted therapies, or radiation. Prominent examples of this include lung cancer, melanoma, and renal cell carcinoma, where ICIs have emerged in the front-line setting [2–4]. There is also a rapidly expanding role for ICIs in the adjuvant, neoadjuvant, and maintenance settings [5–7].

**Fig. 1:**
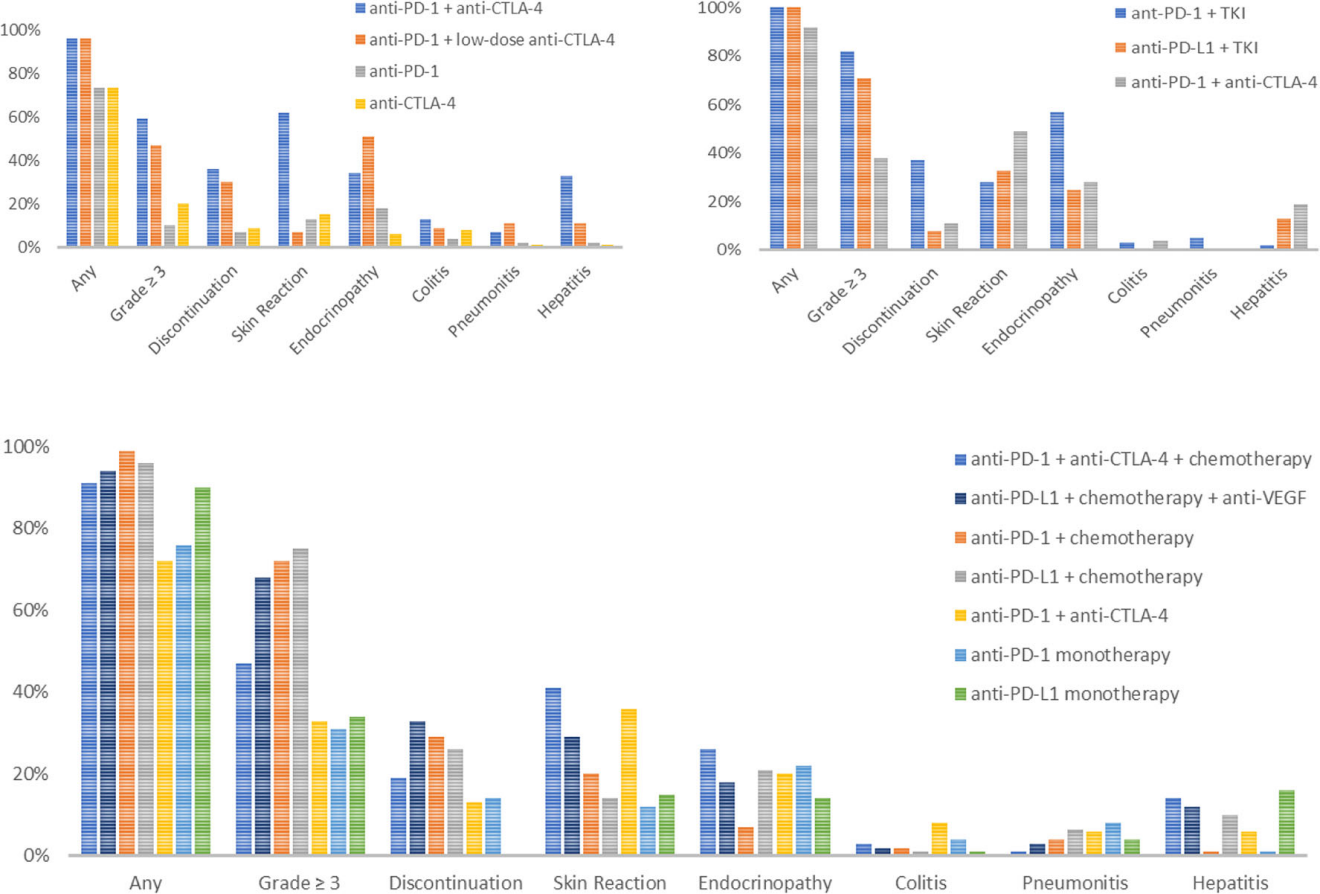
Treatment-related adverse events for select clinical trials in cutaneous melanoma (**A**), renal cell carcinoma (**B**), and non-small cell lung cancer (**C**).

Unfortunately, the cost of such success has been a concomitant increase in the frequency of immune-related adverse events (irAEs) [8]. irAEs are common, problematic, and can be a barrier to further cancer-directed therapies. Figure 1 shows the frequency of treatment-related adverse events (TRAEs) and select irAEs in clinical trials using ICIs in the front-line setting [3, 4, 9–19]. The rate of TRAEs differs between cancer types, the dose of the ICI, the use of dual checkpoint blockade (e.g. anti-CTLA-4 combined with anti-PD-1) versus monotherapy with anti-PD-1, anti-PD-L1, or anti-CTLA-4, and in combination with other therapies, such as chemotherapy, anti-VEGF, or tyrosine-kinase inhibitors (TKIs). In general, the rates and severity of TRAEs increase with combination therapy. There is also an increased rate of treatment discontinuation, ranging between 7 and 14% for ICI monotherapy and 11–36% for anti-PD-1 and anti-CTLA-4 combination therapy [9–12, 15]. These rates are higher when a TKI or chemotherapy is added [4, 18].

Fortunately, we now have effective therapies to treat irAEs. Skin rash of mild severity can often be controlled with topical therapy alone [20], anti-diarrheal medications can be used for mild colitis/diarrhea [21], and many of the endocrinopathies can be treated with hormone replacement [22]. As a result, there is an increasing number of patients who experience irAEs of mild severity that can continue ICIs without a period of discontinuation. Systemic immunosuppressive therapies can be used for the more severe and/or disabling irAEs, often with a period of discontinuation [23]. Corticosteroids are most commonly used in this setting.

This article will review the current knowledge and understanding of the how, when, and who to rechallenge with ICIs after a period of discontinuation due to toxicity. Like any treatment decision, the decision to rechallenge with ICIs will depend on the risk/benefit ratio. The focus of this article will be on what is known about rechallenge risk, ways to mitigate this risk, and a suggested approach to weighing the risks and benefits. Additional guidance on the acute management of common irAEs has previously been published [23–26].

## Rechallenge with immune checkpoint inhibitor(s) after irAE

### Restarting the same immune checkpoint inhibitor(s)

The data that is currently available in the rechallenge setting is all retrospective in nature. No prospective clinical trials have been initiated or published on this topic in the National Institutes of Health registry [27]. The highest level of evidence to date comes from a systematic review and meta-analysis by Zhao et al. (2021) [28•]. Results from this publication and a multitude of cohort studies will be discussed in this section. Most of these studies provide analysis of combined tumor types (referred to as “mixed histology” hereafter) and include different ICI treatment regimens, with a few exceptions that focus on non-small cell lung cancer (NSCLC), renal cell carcinoma (RCC), colorectal cancer (CRC), or melanoma. Here, we have attempted to resolve trends in irAE frequency and type among the different histologies and ICI regimens. Results from select studies are shown in Table 1 [29–41].

**Table 1:** Retrospective studies evaluating irAEs after ICI rechallenge

Rechallenge type	Study	Cancer type	Patients rechallenged	ICI(s)	irAE-2, *n* (%)	irAE-2 grade ≥3, *n* (%)	Same irAE, *n* (%)	Discontinuation rate^a^	ORR	DCR
Restart ICI(s)	Allouchery et al.^b^	Mixed	180	142 anti-PD-1; 9 anti-PD-L1; 11 anti-CTLA-4; 18 anti-PD-1 + anti-CTLA-4	70 (39)	27/180 (15)	52/180 (29)	47/180 (26)	NR	NR
	Bhatlapenumarthi et al.	Mixed	27	25 anti-PD-1; 2 anti-PD-L1	9 (33)	NR	7/27 (26)	NR	NR	NR
	Dolladille et al.	Mixed	60	anti-PD-(L)1 + anti-CTLA-4	NR	NR	18/60 (30)	NR	NR	NR
			370	anti-PD-(L)1	NR	NR	105/370 (28)	NR	NR	NR
			22	anti-CTLA-4	NR	NR	7/22 (32)	NR	NR	NR
	Kartolo et al.^b^	Mixed	40	28 anti-PD-1; 2 anti-CTLA-4; 5 anti-PD-1 + anti-CTLA-4; 5 ICI + chemotherapy	31 (78)	NR	19/40 (48)	8/40 (20)	NR	NR
	Simonaggio et al.^b^	Mixed	40	26 anti-PD-1; 5 anti-PD-L1; 4 anti-PD-1 + anti-CTLA-4; 4 anti-PD-(L)1 + other ICI; 1 other ICI	22 (55)	13/40 (33)	17/40 (43)	NR	NR	NR
	Morse et al.	CRC	25	anti-PD-1 + low-dose anti-CTLA-4	14 (56)	6/25 (24)	NR	NR	NR	NR
	Mouri et al.	NSCLC	21	anti-PD-1	15 (71)	1/21 (5)	9/21 (43)	NR	15/20^c^ (75)	18/20^c^ (90)
	Niki et al.	NSCLC	11	anti-PD-1	5 (45)	0 (0)	NR	0 (0)	3 (50)	4 (67)
	Santini et al.	NSCLC	38	24 anti-PD-(L)1; 14 anti-PD-(L)1 + anti-CTLA-4	20 (52)	10/38 (26)	10/38 (26)	NR	5/38 (13)	33/38 (87)
			8	anti-PD-(L)1 + anti-CTLA-4	4 (50)	NR	NR	NR	NR	NR
	Alaiwi et al.	RCC	36	15 anti-PD-(L)1; 11 anti-PD-1 + anti-CTLA-4; 10 anti-PD-(L)1 + anti-VEGF/other	18 (50)	7/36 (19)	6/36 (17)	10/36 (28)	6/35^c^ (17)	30/35^c^ (86)
De-escalation	Dolladille et al.	Mixed	25	anti-PD-1 + anti-CTLA-4 → anti-PD-(L)1	15 (60)	NR	11 (44)	NR	NR	NR
			11	anti-PD-1 + anti-CTLA-4 → anti-CTLA-4	4 (36)	NR	2 (18)	NR	NR	NR
	Pollack et al.	Melanoma	80	anti-PD-1 + anti-CTLA-4 → anti-PD-1	40 (50)	14/80 (18)	14/80 (18)	24/80 (30)	56/80 (70)	71/80 (89)
	Santini et al.	NSCLC	6	anti-PD-1 + anti-CTLA-4 → anti-PD-(L)1	(54)	NR	NR	NR	NR	NR
Class switch	Abu-Sbeih et al.	Mixed	64	anti-CTLA-4 → anti-PD-(L)1	NR	NR	17/64 (27)	NR	NR	NR
			8	anti-PD-(L)1 → anti-CTLA-4	NR	NR	7/8 (88)	NR	NR	NR
	Menzies et al.	Melanoma	67	anti-CTLA-4 → anti-PD-1	25 (37)	14/67 (21)	2/67 (3)	8/67 (12)	27 (40)	NR

*CRC*, colorectal cancer; *DCR*, disease control rate; *ICI*, immune checkpoint inhibitor; *irAEs*, immune-related adverse events; *irAE-2*, irAE after rechallenge; *NSCLC*, non-small cell lung cancer; *NR*, not reported; *ORR*, overall response rate; *RCC*, renal cell carcinoma

^a^Discontinuation rate due to toxicity

^b^Did not include grade 1 irAE

^c^One patient was not evaluable

In mixed histology studies, the risk of developing any irAE after restarting the same regimen was 33–78% [38, 40]. In the largest study that compared anti-PD-(L)1, anti-CTLA-4, and combination therapy, anti-CTLA-4 rechallenge had a slightly higher rate of the same irAE recurring (32%) vs combination therapy (30%) vs anti-PD-(L)1 alone (28%) [39]. It is important to note that trends between different cancer types may be obscured in mixed histology studies. In one study, patients with melanoma had higher rates of second irAEs (47%), followed by NSCLC (36%), and RCC (11%) [41]. Other NSCLC studies have reported a rate of second irAEs between 45 and 71% [33–35], and the single RCC study reported an irAE rate of 50% [32]. These differences in irAE frequency are at least partially explained by variation in grading and reporting, as some studies did not include grade 1 events.

The risk of developing the same irAE in mixed histology studies after restarting the same ICI(s) was 26-48% [37, 38, 40]. The risk of developing a new irAE was 13-30% [37, 41]. Notably, among patients who did experience a second irAE, the majority (61–78%) experienced a recurrence of the same irAE that initially led to ICI discontinuation [38, 40]. Regimens including anti-CTLA-4 were associated with a higher risk of the same irAE recurring [39].

In NSCLC, the risk of developing any irAE after restarting the same ICI(s) was 50–71% [33, 35]. The majority of patients had a recurrence of the same irAE (50–60%) with the remaining 40–50% of patients experiencing a new irAE. The objective response rate (ORR) ranged from 13 to 75%, while the disease control rate (DCR) was 67–90% [33–35]. This is consistent with the pooled ORR and DCR reported in the meta-analysis of 43.1% and 71.9%, respectively [28•].

There was one cohort of RCC patients who restarted the same ICI(s) [32]. The rate of second irAEs was 50%, 33% were recurring irAEs. This is inconsistent with the other studies that reported rates of 61–78%, and may suggest that RCC patients are more likely to develop new irAEs with rechallenge, although more studies are needed to compare these data [38, 40]. The ORR in this cohort was 17% and the DCR was 86% [32]. The ORR and DCR for RCC was similar to what was reported in cohorts with different cancer types.

In the only colorectal cancer (CRC) study available to date, patients mismatch repair-deficient CRC retreated with anti-PD-1 and anti-CTLA-4 combination had a second irAE rate of 56%, 43% of which were grade ≥ 3 [36]. The rates of recurrent versus new irAEs were not reported in this study. Likewise, no data was provided on the ORR or DCR.

The most common recurrent irAEs across studies were colitis (37–60%), arthritis/arthralgias (45–83%), skin reaction (38%), pneumonitis (20–34%), hepatitis (29–60%), and neutropenia (66.6%) [37, 39]. Patients with gastrointestinal irAEs were more likely to have recurrent grade ≥ 2 irAEs after rechallenge [41]. Similarly, a meta-analysis reported that gastrointestinal irAEs were associated with a higher recurrence of high-grade irAEs [28•]. Endocrinopathies were less likely to recur [39, 41]. This is distinct from irAEs seen with initial ICI treatment, which are most commonly dermatologic (7–62%) and endocrine (6–57%), followed by gastrointestinal, hepatic, and pulmonary irAEs (Fig. 1) [42•, 43•, 44•]. Differences in the first versus second instance of endocrine irAEs may be attributed to the fact that patients with initial endocrinopathies may be on active hormone replacement at the time of rechallenge.

Of the patients who experienced a second irAE, up to 33% experienced grade ≥ 3 events, with a pooled incidence of 12% in the meta-analysis [28•,^37^]. This range was similar to the percentage of patients who had initially experienced grade ≥ 3 irAEs [28•]. None of the second irAEs was more severe than the first irAE, suggesting that rechallenge with the same regimen may be safe [37].

In conclusion, upon rechallenge with the same ICI regimen, it can be expected that between 33 and 78% of patients may experience a subsequent irAE, the majority of which are likely to be the same irAE [38, 40]. This range is consistent with the largest meta-analysis to date, which reported an all-grade irAE rate with rechallenge of 34.2% [28•]. Of note, patients treated with concurrent chemotherapy or TKI were excluded in the meta-analysis. An increased likelihood of recurrence may be expected for patients who experience an initial gastrointestinal irAE [28•, 39, 41]. Less than a third of the second irAEs can be expected to be grade ≥ 3, and it is unlikely that a second irAE will be more severe than the first; this is consistent with the meta-analysis, which reported a high-grade irAE rate of 11.7% [28•]. These rates may be higher in patients treated with regimens including single-agent or combination anti-CTLA-4 [39]. This conclusion may inform clinical decision-making for patients who are unwilling or unable to tolerate the same irAE after rechallenge, especially for patients with an initial gastrointestinal irAE; for these patients, it may be useful to recommend a de-escalation approach or treatment cessation.

### De-escalation

De-escalation, defined hereafter as a change from ICI combination therapy to ICI monotherapy, is another approach that can be considered after an irAE has occurred. In three de-escalation studies, resumption of anti-PD-(L)1 monotherapy had a higher rate of overall and recurrent irAEs (50–60% overall, 18–44% recurrent) when compared to resumption of anti-CTLA-4 alone (36% overall, 18% recurring) [31, 33, 39]. This is in contrast with the meta-analysis, which reported that anti-PD-(L)1 rechallenge was associated with a lower recurrence of all-grade irAEs [28^•^]. Patients with melanoma who switched from combination anti-PD-1 and anti-CTLA-4 to anti-PD-1 monotherapy reported overall irAE rates of 50%, with 18% grade ≥ 3 and 18% having the same irAE [31]. Interestingly, colitis seemed especially unlikely to recur in this cohort, with only 2 patients (6%) experiencing a recurrence; this contrasts with other studies above in which colitis has been reported to recur more frequently with rechallenge of the same ICI(s).

### Class switch

Switching from anti-PD-(L)1 to anti-CTLA-4, or vice versa, is another option to consider. In one study of immune-mediated diarrhea and colitis (IMDC), patients switched from anti-PD-(L)1 to anti-CTLA-4 had a recurring irAE rate of 88%, compared to a rate of 27% in a cohort switched from anti-CTLA-4 to anti-PD-(L)1 [29]. Patients with melanoma who switched from anti-CTLA-4 to anti-PD-(L)1 had an overall irAE rate of 37%, the majority (56%) of which were grade ≥ 3, but only 2 irAEs were recurring (3%; arthritis and colitis); this low recurrence rate is unique among the summarized studies [30]. For patients initially treated with anti-PD-(L)1 ICIs, rechallenge with anti-CTLA-4 antibodies had a significantly higher incidence of all-grade irAEs than anti-PD-(L)1 antibody rechallenge, further supporting a restart approach over a class switch for patients with initial anti-PD-(L)1 treatment [28•]. Although there may be distinct differences in irAE rates between cancer types, these may be confounded by the treatment regimen given—i.e., anti-CTLA-4 is more likely to be used in melanoma or RCC vs NSCLC; therefore, the differences in irAE rates may be at least partially attributed to differences in regimen used between cancer types.

### Chemotherapy and TKI

Can patients be rechallenged with ICIs in combination with chemotherapy or other drugs? At this time there is very little data on the success of such approaches, all of which is retrospective in nature. For the combination of ICI with chemotherapy, only one study was identified [38]. This study included 6 patients with advanced cancer who were initially treated with an ICI and chemotherapy and then developed grade ≥ 2 irAEs. Five of the patients were then rechallenged with an ICI and chemotherapy. Four of these five patients developed an irAE (80%). In the total rechallenge cohort (*n*=40), including patients who did not receive chemotherapy, there was a 78% chance of subsequent irAE and a 42% chance of recurrent irAE. The authors concluded that it is relatively safe to rechallenge patients with ICIs. However, it is our opinion that more data is needed to draw any meaningful conclusion on the safety and efficacy of rechallenge with combination ICI and chemotherapy.

ICIs have also been combined with tyrosine-kinase inhibitors (TKIs) in the rechallenge setting. Three separate studies identified a total of 16 patients treated with the combination of ICI and TKI, all of whom had metastatic renal cell carcinoma [32, 45, 46]. Two of the studies reported the rate of irAEs upon rechallenge, which ranged from 38-60% [32, 45]. Only one study reported ORR and DCR, which were 60% and 80% respectively [45]. While it is difficult to draw any strong conclusion from these studies due to the small sample sizes, it is notable that the risk of irAEs upon rechallenge with ICI and TKI does not appear to be any higher than that observed in patients rechallenged without a TKI. The severity of irAEs also did not exceed grade 3 toxicity in the two studies that reported this data [32, 45].

These results are promising, but larger studies are needed to confirm these findings. There will also be interest in studying the combination of ICI and TKI rechallenge across additional cancer types. For example, advanced endometrial cancer, where the combination of ICI and anti-VEGF TKI is now approved in the second-line setting [47, 48].

### Rechallenge with concurrent immunosuppression

Our current data on the prevention of irAEs is limited to outcomes observed from case series and retrospective studies. Table 2 summarizes these findings [29, 49, 50]. First-line management of irAEs often involves the use of corticosteroids. Their use in the preventive setting remains to be fully elucidated. In one meta-analysis of 16 studies, patients receiving ICI and steroids for any reason were at increased risk for death (HR = 1.54; 95% CI 1.24–1.91) and disease progression (HR 1.34; 95% CI 1.02–1.76) compared to patients not receiving steroids [51]. The risk of death was the highest in the subset of patients who were receiving corticosteroids for palliative indications (HR 2.5; 95% CI 1.41–4.43) with outcomes likely owing to the overall poor-prognosis of this subgroup. On the other hand, steroids used to mediate irAEs were not associated with worse overall survival (HR 1.08, 95% CI 0.79-1.49).

**Table 2:** Studies evaluating irAEs after ICI rechallenge with concurrent IST

Study	Cancer type	ICI(s)	irAE-1	Patients rechallenged with IST	IST, n (%)	irAE-2, *n* (%)	Recurrent irAE, *n* (%)	New irAE, *n* (%)	Recurrent irAE grade ≥3, *n* (%)	Discontinuation rate^a^	ORR	DCR
Kim et al.	Melanoma	anti-PD-1 → anti-CTLA-4	Arthritis	1	Tocilizumab	NR	0 (0)	NA	NA	NA	NR	NR
Abu-Sbeih et al.	Mixed	47 anti-CTLA-4; 79 anti-PD-(L)1; 41 ICI combination	Colitis	113	Corticosteroid, 113 (100) Infliximab or Vedolizumab, 24 (14)	NR	47 (42)	NR	NR	NR	NR	NR
Badran et al.	Mixed	1 anti-CTLA-4; 2 anti-PD-1; 2 anti-CTLA-4 + anti-PD-1	Colitis	5	Infliximab, 5 (100)	3 (60)	1 (20)	2 (40)	2 (40)	1/5 (20)	20%	80%

*DCR*, disease control rate; *ICI*, immune checkpoint inhibitor; *irAEs*, immune-related adverse events; *irAE-1*, irAE before rechallenge; *irAE-2*, irAE after rechallenge; *IST*, immunosuppressive therapy; *NA*, not applicable; *NR*, not reported; *ORR*, overall response rate

^a^Discontinuation rate due to toxicity

Cytokine inhibitors are therapeutic options when immune-mediated toxicities are refractory to corticosteroids. Their role in irAE prevention were first studied in colon cancer mouse models where the concurrent use of TNF inhibitors with dual anti-CTLA-4 and PD-1 therapy improved autoimmune colitis and enhanced anti-tumor efficacy [52]. In one case series, five patients were treated concomitantly with infliximab in combination with anti-CTLA-4 and PD-(L)1 therapy or PD-1 monotherapy as secondary prevention for autoimmune colitis [49]. Doses of ICI administered along with infliximab ranged from 2 to 12 doses. In 4 out of 5 patients who had repeat endoscopies 3–4 months after concurrent infliximab and ICI therapy, no acute inflammation was observed endoscopically to suggest colitis recurrence. All but one patient in this case series had disease progression following anti-TNFα and ICI therapy. The incidence of recurrent colitis was 20%. In comparison, the incidence of recurrent IMDC was 34% in one study that examined patients rechallenged with ICI alone [29].

Combining TNFα inhibitors with ICIs is a potential strategy for irAE prevention. Contrary to the efficacy observed in mouse models, anti-TNFα with or without steroids for the treatment of steroid-refractory immunotherapy-related enterocolitis was associated with a significantly decreased OS compared to patients who received steroids alone with a median OS of 17 and 27 months, respectively (HR 1.61; 95% CI, 1.03-1.51) [49]. A phase 1b study, the TICIMEL trial, is currently underway to evaluate the safety, tolerability and clinical outcomes of combined anti-TNFα and ICI therapy in patients with advanced melanoma [53].

Administration of other cytokine inhibitors in addition to anti-TNFα agents have been reported including tocilizumab, an anti-IL6 receptor monoclonal antibody. One case report of a patient with advanced melanoma and refractory Crohn’s disease receiving concurrent therapy with pembrolizumab and tocilizumab experienced a delay in Crohn’s disease exacerbation for at least 16 weeks while retaining an antitumor response [54]. Another small study included 2 patients who received tocilizumab prophylactically with no irAE occurrence [55].

Targeting cytokines may represent a mechanism of preventing irAEs with ongoing studies investigating the use of cytokines as predictive biomarkers for identifying patients at risk for irAEs. For example, increased serum IL-17 concentrations were found in metastatic melanoma patients treated with ipilimumab who developed immunotherapy-related colitis [56, 57]. Serum IL-17 concentrations decreased to levels equivalent to that of patients without colitis following the resolution of colitis, suggesting the correlation between cytokine concentrations and irAE disease status [56–58]. Whether specific cytokine inhibition based on cytokine profiling prevents irAEs in patients on ICIs still has yet to be determined.

Additionally, vedolizumab, a monoclonal antibody that targets α4β7 integrin and inhibits gastrointestinal lymphocyte trafficking, was studied in a small subset of patients receiving concurrent ICI following autoimmune colitis resolution [29, 59]. One out of 8 patients treated with vedolizumab experienced a colitis recurrence while recurrence occurred in 3 out of 6 patients who did not receive vedolizumab [29]. For steroid-refractory IMDC, one retrospective study found that vedolizumab was associated with superior survival outcomes compared to infliximab [60].

## Suggested approach to ICI rechallenge

The decision to rechallenge a patient with ICI(s) after toxicity is complex and should take into account the risks and benefits of retreatment, as well as the patient’s preference to resume therapy. The patient may have a strong opinion about ICI rechallenge, particularly if the toxicity was severe and/or had a significant impact on the quality of life. It therefore behooves the treating clinician to elucidate any concerns the patient may have about retreatment, and to actively engage in shared decision making whenever possible. Once a discussion has been initiated, the patient and provider will want to discuss the risks and benefits of retreatment that are categorized below, noting that additional questions/concerns may be of importance.

One of the first questions to consider is whether alternative treatment options exist that have a higher chance of benefiting the patient. While ICI is a useful treatment modality, targeted therapies, chemotherapy, and even local therapies (in the setting of oligoprogressive or oligometastatic disease) have shown efficacy in the second-line and beyond for many cancer types. Careful consideration should be given to the rates of response and toxicity between alternative agents.

As shown in Table 1, the ORR and toxicity of ICI rechallenge can be highly variable, even within the same tumor type. The DCR has been more consistent with ICI rechallenge across tumor types and drug class, ranging between 86 and 90% [31–33, 35], and this should be considered when stable disease is an acceptable outcome. More recently, a systematic review and meta-analysis reported a pooled ORR and DCR after rechallenge of 43.1% and 71.9%, respectively across all cancer types [28•]. There have been no clinical trials to date to provide an accurate comparison of efficacy with alternative treatments. In general, we recommend an alternate treatment over ICI rechallenge when there is a reasonable chance the patient will have a better response to the alternative choice. When the alternatives to ICI rechallenge have a low efficacy rate, the focus should next be on the safety of ICI rechallenge.

Safety will depend on a number of factors including, which organ experienced the initial irAE, the severity of the irAE and how difficult it was to manage, patient-specific factors, and whether the patient will be rechallenged with ICI alone or in combination with other agents. The target organ and severity of the initial irAE should be carefully considered due to the risk of recurrent irAEs, which is reportedly between 17 and 88% [29, 32]. The risk of any irAE is even higher when accounting for new irAEs. A systematic review and meta-analysis reported a pooled incidence of all-grade and ≥ grade 3 irAEs after rechallenge of 34.2% and 11.7%, respectively across all cancer types [28•].

The target organ of the initial irAE becomes particularly relevant when toxicity to that organ has a high risk of morbidity/mortality (e.g. cardiac or CNS toxicity). In these instances, it is generally advised to avoid rechallenge for any toxicity above grade 1 [24]. Conversely, if the irAE manifests as an endocrinopathy, even severe toxicities can be managed with hormone replacement while the ICI is continued. General practice recommendations include holding ICI treatment for toxicities that are grade ≥ 2 until the toxicity has reverted to grade ≤ 1 and permanent discontinuation of ICI(s) for grade 4 toxicities. Additional guideline recommendations on ICI rechallenge for each organ system have been published [24, 26].

Patient-specific factors include, comorbidities, performance status (PS), and expected longevity, each of which can influence the decision to rechallenge. The link between autoimmune disorders and irAEs is well-known, but emerging data has also shown an increased risk of immune-mediated pneumonitis with pre-existing pulmonary disease [61, 62]. Similar relationships may exist between the irAE target organ and the underlying health/function of that organ. This remains an area of active investigation.

Poor PS, defined as Eastern Cooperative Oncology Group (ECOG) PS ≥ 2, has been associated with decreased efficacy of ICIs. Multiple retrospective studies have shown shorter progression free survival (PFS) and OS when the ECOG PS was ≥ 2 [63–67]. ORR was also lower across cancer types, with the exception of advanced urothelial cancer. Patients with an ECOG PS ≥ 2 were also less likely to be referred to hospice and more likely to die in the hospital when they were treated with ICIs [66, 67]. Based on the above, it would be expected that the efficacy of ICI rechallenge would be significantly less if the patient’s PS is ECOG ≥ 2. Many of these patients will qualify for hospice, and the treating physician should consider what the patient’s goals are for end-of-life care. A frank discussion of these goals may impact the decision to rechallenge with ICI(s).

Finally, once a decision has been made to rechallenge, the focus should shift to the when, what, and how. The “when” will be determined by the severity of the initial irAE and the urgency of treatment. Guideline recommendations include holding ICI treatment for toxicities that are grade ≥ 2 until the toxicity has reverted to grade ≤ 1 with few exceptions [24]. If the patient requires urgent treatment, the treating clinician should re-evaluate whether ICI rechallenge is the best available option and if local therapies can be used either before or concurrently with ICI(s).

What ICI to use, either as monotherapy or in combination, should also be carefully considered. There is no randomized data in the rechallenge setting to inform this decision, and even the limited retrospective studies have not done a direct comparison between the different approaches (de-escalation, ICI restart, or class switch). In general, we favor a de-escalation approach when dual-checkpoint blockade was the original treatment modality. This is supported by data in the front-line setting, where multiple phase 3 clinical trials have shown higher rates of irAEs and treatment discontinuation with ICI combinations compared to monotherapy [3, 16]. There is again limited data to compare the efficacy and toxicity of a class switch approach, but the toxicity profile appears to be similar when switching from anti-CTLA-4 to anti-PD-(L)1 compared to a restart of anti-CTLA-4 ICI [29, 30, 39]. On the other hand, switching from anti-PD-(L)1 to anti-CTLA-4 had a much higher toxicity rate [29]. This approach is not recommended at this time.

The “how” refers to the decision of whether to add or continue immunosuppression with the ICI(s), general oversight, and monitoring recommendations. At the present time, there is insufficient data to recommend concurrent immunosuppression with ICI rechallenge. However, multiple small-scale studies suggest there may be improved safety with this approach, and further research is warranted [29, 49, 50]. Ideally, every rechallenge decision should include input from a multidisciplinary team, and a systematic process should be established to review individual cases whenever possible. Close monitoring is recommended. If a subsequent irAE occurs, permanent discontinuation is advised in most instances.
